# Direct amidation of non-activated phenylacetic acid and benzylamine derivatives catalysed by NiCl_2_

**DOI:** 10.1098/rsos.171870

**Published:** 2018-02-21

**Authors:** Lidan Cheng, Xiaoping Ge, Longjiang Huang

**Affiliations:** 1 College of Chemical Engineering, Qingdao University of Science and Technology, Qingdao 266042, People's Republic of China; 2 State Key Laboratory of Bioactive Substance and Function of Natural Medicines, Institute of Materia Medica, Chinese Academy of Medical Sciences and Peking Union Medical College, Beijing 100050, People's Republic of China

**Keywords:** direct amidation, nickel dichloride, phenylacetic acid derivatives, benzylamine derivatives

## Abstract

This paper describes an eco-friendly and efficient direct amidation of benzylamine and phenylacetic acid derivatives in the presence of 10 mol% NiCl_2_ as catalyst without any drying agent. For the different phenylacetic acid and benzylamine derivatives, the direct catalysed amidation gave moderate-to-excellent yields in toluene. The steric and electronic effects of substituent groups on the phenyl ring of acid were crucial to the yields of the direct amidation. The catalyst NiCl_2_ can be recycled three times without loss of activity.

## 1. Introduction

Amide bonds, as the key bonds of proteins, are widespread in drugs [1–3], polymers, biomacromolecules and food additives [4–8]. In a survey, amide bonds were found in two-thirds of drug candidates and present in one-fourth of all drugs [9,10]. Furthermore, the high stability of amide bonds has led to their extensive applications in materials such as nylon and artificial silk.

Current industrial processes for forming amide bonds include two main strategies: one is the use of stoichiometric amounts of expensive coupling reagents such as carbodiimides or phosphonium or uronium salts to activate and condense the carboxylic acid with amine [11]. The other is the activation of carboxylic acid as acyl halide, acyl imidazole, acyl azide, anhydride or active ester followed by aminolysis [12,13]. Both methods generate large amount of wasteful by-products, therefore increasing the difficulty and cost of isolating the desired amide product [14].

Direct catalysed amidation of carboxylic acid with amine is highly attractive from the industrial perspective as it would lead to cost-effective and atom-economic processes with water as the only by-product. The challenge of catalysed amidation methods is that acid–base reaction may occur between the acid and the amine. The salt formation can be overcome by elevating the reaction temperature and amides can be obtained in moderate-to-good yields depending on the substances in use. However, high temperature (greater than 180°C) [15] used in some direct amidation is not suitable for highly functionalized or sensitive substances.

In the last 10 years, catalytic direct amidations catalysed by organo-boron derivatives have emerged, and lots of catalytic protocols have been discovered so far for the direct amidation of carboxylic acids and amines, and works on these topics were already well reviewed by Figueiredo *et al.* [16]. Although underdeveloped, metal-catalysed direct amidations have received increasing attention in the past few years, most of this attention being focused on early transition metal complexes involving titanium complexes such as Ti(OiPr)_4_, Ti(OBn)_4_ and TiCl_4_ [17]; zirconium salts or complexes such as ZrCl_4_, Zr(OEt)_4_ and Zr(Cp)_2_Cl_2_ [18,19]; Hf(Cp)_2_Cl_2_ [20] and others [21,22]. Some of them showed good efficiency for amidations; however, the drawbacks of these metal catalysts are their insensitivity to water and air or difficulty to be recycled.

Using nickel as catalyst has received extensive attention because of its low cost, good reactivity, good stability under air and water and nontoxicity. Nickel was applied in different types of reactions such as reduction [23], coupling [24–28] and others [29–31].

As part of our continuing programme to develop recyclable catalysts for direct amidation, we herein present an efficient and green nickel catalyst for the direct amidation of phenylacetic acid and benzylamine. To the best of our knowledge, this is the first report of nickel catalysed direct amidation of acid and amine.

## 2. Results and discussion

**Figure 1.**
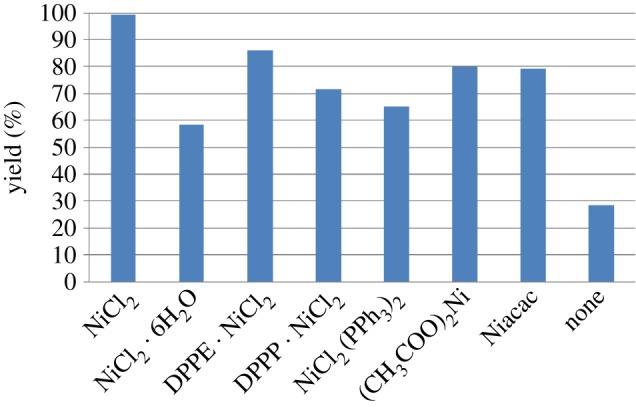
The amidation of phenylacetic acid with benzylamine catalysed by nickel metal. Reaction conditions: phenylacetic acid 1a (2 mmol), benzylamine (2.4 mmol), catalyst (10 mol%), toluene (20 ml), 110°C, 20 h.

Phenylacetic acid and benzylamine were chosen as model substrates to investigate the performance of the different catalysts including NiCl_2_, NiCl_2_ · 6H_2_O, DPPE · NiCl_2_, DPPP · NiCl_2_, NiCl_2_(PPh_3_)_2_, (CH_3_COO)_2_Ni and Ni(acac)_2_. To verify if the amidation reaction was indeed catalysed by nickel catalysts, control experiment was performed in the absence of the catalyst. To our delight, we found that all the examined nickel compounds did catalyse the amidation reaction. Figure 1 shows the performance of different nickel catalysts used for the amidation of phenylacetic acid and benzylamine in toluene at 110°C.

The catalytic activities of the different nickel catalysts were NiCl_2_ > DPPE · NiCl_2_ > (CH_3_COO)_2_Ni >Ni(acac)_2_ > DPPP · NiCl_2_ > NiCl_2_(PPh_3_)_2_ > NiCl_2_ · 6H_2_O > none. Usually, homogeneous metal compl-exes show higher catalytic activity than the heterogeneous metal halides [32]; interestingly, in our experiment, NiCl_2_ was found to be the most efficient catalyst among the nickel metals and its complexes.

The reaction proceeded smoothly in a sealed vessel without any drying agent such as molecular sieves. The result shows that the equilibrium is favourable without the need to remove water and this phenomenon was firstly demonstrated by Allen *et al.* in 2012 [33]. The process without any drying agent made the separation of the product and the recovery of the catalyst easier.

The influences of different solvents and reaction time on the direct amidation of phenylacetic acid with benzylamine catalysed by NiCl_2_ were investigated (table 1).

**Table 1.** Optimization of the reaction conditions using different solvents.

entry	solvent^a^	temperature (°C)^a^	yield^b^ (%)-10 h	yield^b^ (%)-20 h
1	Et_2_O	30	nd	nd
2	DCM	40	nd	nd
3	DCM	60	nd	nd
4	THF	70	nd	nd
5	Et_2_O : THF = 1 : 1	40	nd	nd
6	toluene	60	nd	Trace
7	toluene	80	12.5	36.6
8	toluene	110	80.0	99.2
9	PhF	80	42.1	60.2
10	MeCN	80	nd	nd
11	DMF	100	nd	nd
12	DMSO	100	nd	nd

^a^Reaction conditions: phenylacetic acid (2 mmol), benzylamine (2.4 mmol), catalyst (10 mol%), solvent (20 ml).

^b^Isolated yields.

The reactions of phenylacetic acid with benzylamine in polar solvents such as DCM, DMF, DMSO, THF and MeCN were sluggish in 10 h, and on prolonging the reaction time to 20 h, the products were still not detected (table 1, entries 1–5 and 10–12). Reactions in non-polar solvents such as PhF and toluene proceeded with low-to-good yields in 10 h; on prolonging the reaction time to 20 h, the reaction proceeded in moderate-to-excellent yields (table 1, entries 7–9) and the best yield (99.2%) was obtained in toluene at 110°C in 20 h (table 1, entry 8).

The catalyst loading was evaluated by using phenylacetic acid and benzylamine as substrates. When the catalyst loading is 10 mol% of the acid, the yield of the amidation is excellent (99.2%, isolated yield). Reducing the catalyst loading to 5 mol% of the acid resulted in lower isolated yield (62.2% isolated yield).) Increasing the catalyst loading to 20 mol% of the acid resulted in similar isolated yield with 10 mol% catalyst loading (98.9% isolated yield). The results showed that 10 mol% catalyst loading is enough for the direct amidation.

With the above knowledge in hand, we investigated the acid **1** and benzylamine **2** scopes of the NiCl_2_-catalysed direct amidation protocol at the same reaction condition. And the results are shown in table 2.

**Table 2.** Synthesis of amide derivatives from carboxylic acids and amines using NiCl_2_ as catalyst.

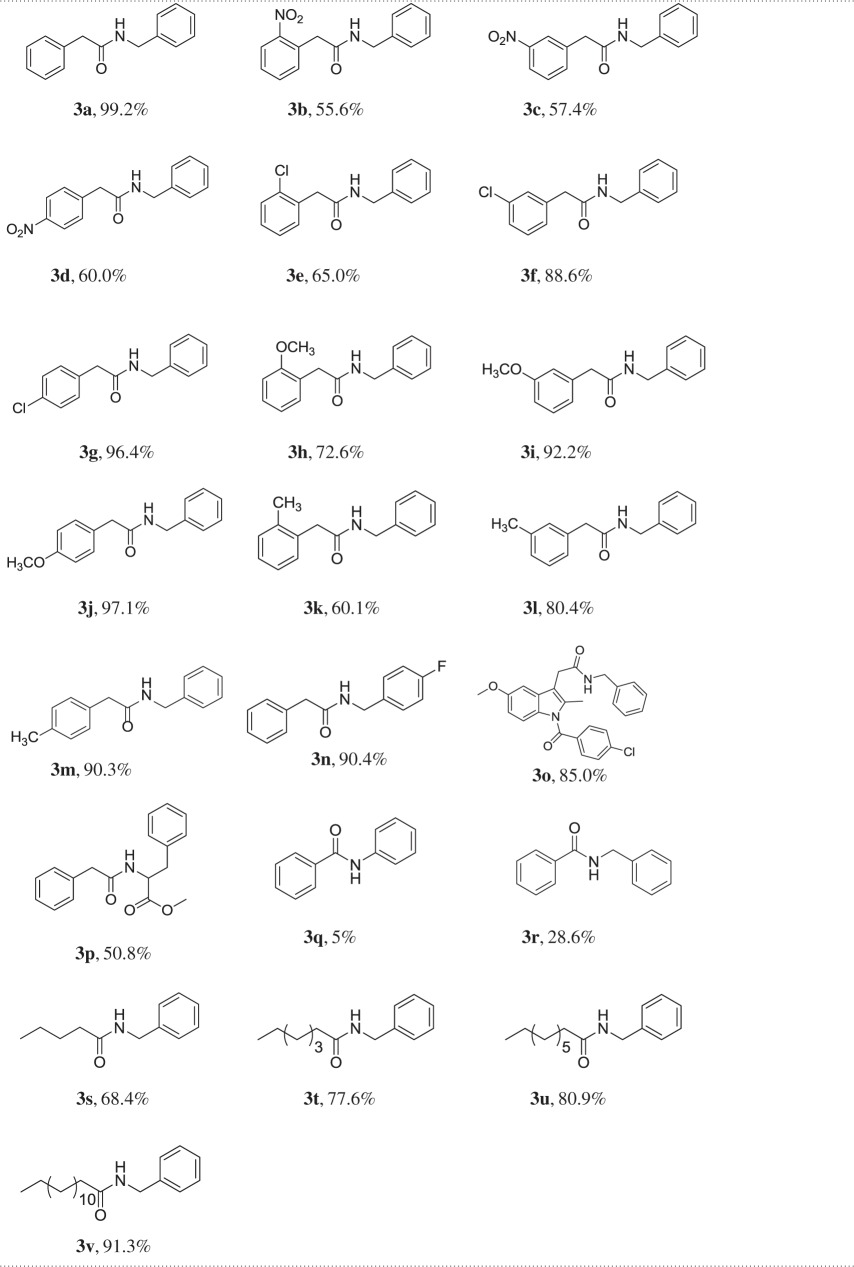


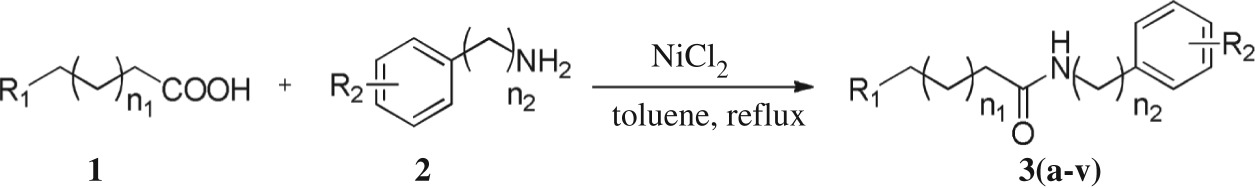


For the different phenylacetic acid derivatives, the direct amidation catalysed by NiCl_2_ worked smoothly. The results showed that substituent groups on the aromatic ring play the crucial role for the direct amidation and the yields are sensitive to the steric and electronic substituent effect of the substituent groups on the aromatic ring.

With the same substituent on the different positions of the aromatic ring, the order of the amidation yield was *para*>*meta*≫*ortho* (table 2, entries **3b–3d, 3e–3g, 3h–3j, 3k–3m**). The ortho substituents with strong steric effect decreased the direct amidation yield (table 2, **3e, 3h, 3k**). With a strong electron-withdrawing group such as nitro group on the *ortho*, *para* and *meta* position of the aromatic ring, the direct amidations catalysed by NiCl_2_ gave moderate yields (55.6%–60.5%, table 2, **3b–3d**). With strong electron-donating substituents such as OMe on the *ortho*, *para* and *meta* position of the aromatic ring, the reaction worked very well with good-to-excellent yields (table 2, **3h–3j**, 72.6–97.1%). For weak electron-withdrawing and electron-donating substituent groups such as Cl and CH_3_, the reaction worked with good-to-excellent yield (table 2, **3e–3g** and **3k–3m**). When using 4-fluorobenzylamine instead of benzylamine, the yield was 90.4% (table 2, **3n**). When structurally more complex nonsteroidal anti-inflammatory drug indomethacin was used as acid substrate, the reaction between indomethacin and benzylamine smoothly produced the corresponding amide in 85% isolated yield (table 2, **3o**). When using the methyl ester of l-phenylalanine as substrate, the racemic product (table 2, **3p**) was obtained in moderate yield. Methyl ester of l-phenylalanine and separately prepared l-**3p** both racemized in our reaction condition. The result is consistent with that reported by Basavaprabhu *et al.* [24]. When using benzoic acid and aniline as the reaction substrates, the amide product was obtained in 5% yield (table 2, **3q**), and using benzylamine instead of aniline as substrate, the reaction yield was 28.6% (table 2, **3r**). When using different chain lengths of fatty acid and benzylamine as substrates, the corresponding amides were obtained in good-to-excellent yields and the increase in chain length enhances the yield of the corresponding amide (table 2, **3s, 3t, 3u** and **3v**).

**Figure 2.**
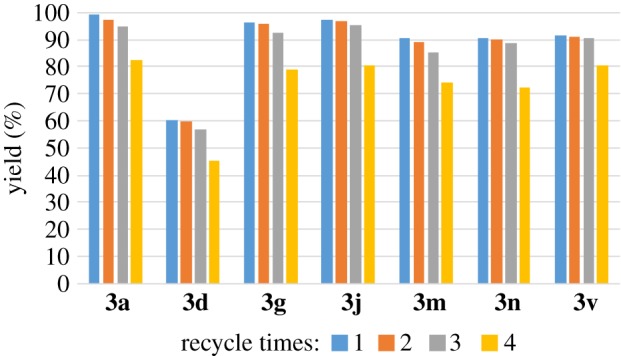
The yield of direct amidation with recycled catalysts.

Catalyst recyclability is an essential aspect of green chemistry. Our best-performance direct amidation catalyst NiCl_2_ was found to be equally effective from fresh up to the third cycle without significant loss of activity as shown in figure 2. In the fourth cycle, the yield decreased obviously because of the loss of the catalyst.

The reversibility of this amidation reaction was tested by reaction compound **3a** with water under the standard reaction condition. In the absence of catalyst, no hydrolysis was seen and with 10 mol% NiCl_2_ only 7% hydrolysis was observed. The results were similar to those reported by Allen *et al.* [33].

**Scheme 1.**
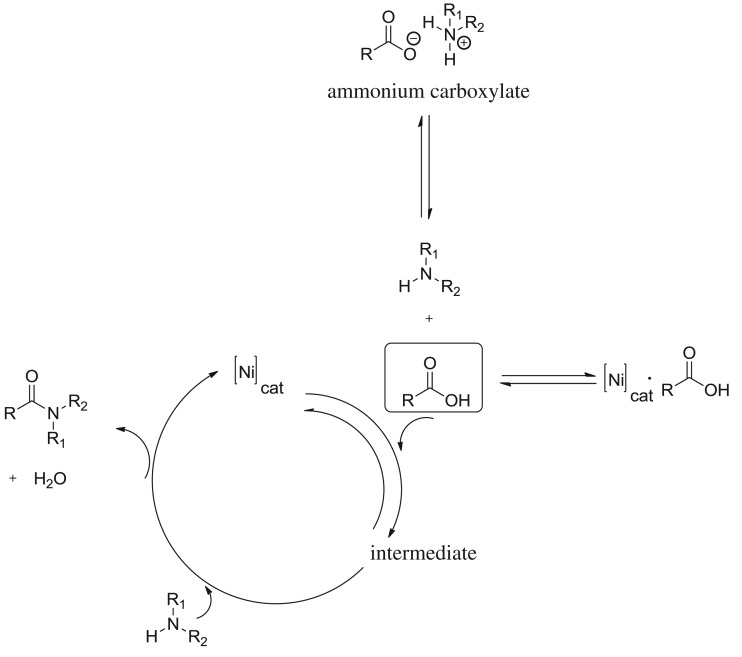
Possible mechanism of amide bond formation catalysed by NiCl_2_.

Helena *et al.* proposed that metal-catalysed direct amidation is not simply Lewis-acid-catalysed by studying ZrCl_4_-catalysed direct amidation [19]. They carried out a control experiment using 20 mol% of HCl (2 M in diethyl ether) as catalyst, resulting in an isolated yield of amide of 8% after 24 h. When compared with the thermal catalyst-free background reaction (13% isolated yield), it clearly demonstrated that HCl is not the active catalyst. In order to study the possible mechanism of NiCl_2_-catalysed direct amidation, we applied the same strategy by a control experiment using 5 mol% of HCl in toluene as catalyst, resulting in an isolated yield of amide of 25% after 24 h, the yield of thermal catalyst-free background reaction being 28.4%. According to the result which was similar to that obtained by Helena *et al.*, we postulated that the NiCl_2_-catalysed direct amidation is not simply Lewis-acid-catalysed amidation. The possible mechanism of NiCl_2_-catalysed amidation is depicted in scheme 1; however, the detailed structure of the intermediate in the catalytic cycle is not yet clear.

## 3. Experimental

General procedure for the preparation of **3a–3v**.

To a solution of acid (2.0 mmol) in toluene (20 ml), NiCl_2_ (10 mol%) was added. The mixture was stirred at 80°C for 10 min, and amine (2.4 mmol) was added to the reaction mixture. After that, the vessel was sealed and the mixture was stirred for 20 h at 110°C, the reaction mixture was cooled to room temperature, filtered and the cake was washed with ethyl acetate to recycle the catalyst, the combined filtrate was washed with HCl (1 mol l^−1^) and then sat. NaHCO_3_. The organic layer was dried over Na_2_SO_4_, filtered and evaporated *in vacuo*. The products were obtained by flash column chromatography.

Characterization data for amides **3a–3v**.

Compound **3a**: White solid (446.4 mg, 99.2%); mp 118–119°C; ^1^H NMR (500 MHz, CDCl_3_) *δ* 7.13–7.22 (m, 11H), 5.66 (brs, 1H), 4.36–4.38 (d, *J* = 5.7 Hz, 2H), 3.59 (s, 2H); HR-MS (ESI^+^) calcd. for C_15_H_16_NO [M + H]^+^: 226.1232. Found 226.1230.

^1^H data were consistent with those reported in the literature [34].

Compound **3b**: White solid (300.2 mg, 55.6%); mp 140–142°C; ^1^H NMR (500 MHz, CDCl_3_) *δ* 8.04 (s, 1H), 7.25–7.62 (m, 9H), 6.09 (brs, 1H), 4.45–4.47 (d, *J* = 5.6 Hz, 2H), 3.88 (s, 2H); ^13^C NMR (125 MHz, CDCl_3_) *δ* 40.83, 43.77, 125.08, 127.43, 127.62 (2C), 128.39, 128.64 (2C), 130.30, 133.43, 133.54, 137.97, 148.85, 168.88; HR-MS (ESI^+^) calcd. for C_15_H_15_N_2_O_3_ [M + H]^+^: 271.1083. Found 271.1083.

Compound **3c**: White solid (309.9 mg, 57.4%); mp 107–108°C; ^1^H NMR (500 MHz, CDCl_3_) *δ* 8.13–8.15 (d, *J* = 8.7 Hz, 2H), 7.23–7.67 (m, 8H), 5.81 (brs, 1H), 4.44–4.46 (d, *J* = 5.6 Hz, 2H), 3.67 (s, 2H); ^13^C NMR (125 MHz, CDCl_3_) *δ* 42.78, 43.87, 122.24, 124.13, 127.64, 127.70 (2C), 128.74 (2C), 129.61, 135.53, 136.81, 137.71, 148.31, 169.27. HR-MS (ESI^+^) calcd. for C_15_H_15_N_2_O_3_ [M + H]^+^: 271.1083. Found 271.1085.

Compound **3d**: White solid (324.0 mg, 60.0%); mp 185–186°C; ^1^H NMR (500 MHz, CDCl_3_) *δ* 8.12–8.15 (s, 1H), 7.15–7.42 (m, 9H), 5.65 (brs, 1H), 4.36–4.38 (d, *J* = 5.6 Hz, 2H), 3.61 (s, 2H); ^13^C NMR (125 MHz, CDCl_3_) *δ* 43.18, 43.93, 123.92 (2C), 127.73 (3C), 128.77 (2C), 130.16 (2C), 137.63, 142.17, 147.18, 168.87; HR-MS (ESI^+^) calcd. for C_15_H_15_N_2_O_3_ [M + H]^+^: 271.1083. Found 271.1082.

Compound **3e**: White solid (336.8 mg, 65.0%); mp 100–102°C; ^1^H NMR (500 MHz, CDCl_3_) *δ* 7.15–7.34 (m, 9H), 5.81 (brs, 1H), 4.37–4.39 (d, *J* = 5.7 Hz, 2H), 3.69 (s, 2H); ^13^C NMR (125 MHz, CDCl_3_), *δ* 41.48, 43.62, 127.38 (2C), 127.45 (2C), 128.60 (2C), 128.96, 129.79, 132.90, 134.56, 138.00, 169.48; HR-MS (ESI^+^) calcd. for C_15_H_15_NOCl [M + H]^+^: 260.0842. Found 260.0848.

Compound **3f**: White solid (486.0 mg, 88.6%); mp 115–116°C;^1^H NMR (500 MHz, CDCl_3_) *δ* 7.16–7.32 (m, 10H), 5.7 (brs, 1H), 4.42–4.43 (d, *J* = 5.7 Hz, 2H), 3.58 (s, 2H); ^13^C NMR (125 MHz, CDCl_3_) *δ* 43.20, 43.67, 127.51 (4C) 128.67 (3C), 129.44, 130.12, 134.65, 136.72, 137.93, 169.98; HR-MS (ESI^+^) calcd. for C_15_H_15_NOCl [M + H]^+^: 260.0842. Found 260.0843.

Compound **3g**: White solid (499.4 mg, 96.4%); mp 146–148°C; ^1^H NMR (500 MHz, CDCl_3_) *δ* 7.158–7.271 (m, 10H), 5.642 (brs, 1H), 4.375 (s, 2H), 3.537 (s, 2H); ^13^C NMR (125 MHz, CDCl_3_) *δ* 42.96, 43.67, 127.55 (3C), 128.67 (2C), 129.07 (2C), 130.68 (2C), 133.21 (2C), 137.93, 170.21; HR-MS (ESI^+^) calcd. for C_15_H_15_NOCl [M + H]^+^: 260.0842. Found 260.0843.

Compound **3h**: White solid (370.3 mg, 72.6%); mp 88–90°C; ^1^H NMR (500 MHz, CDCl_3_) *δ* 7.31–7.92 (m, 7H), 7.02–7.12 (m, 2H), 6.16 (brs, 1H),4.55–4.57 (d, *J* = 5.8 Hz, 2H), 3.94 (s, 3H), 3.78 (s, 2H); ^13^C NMR (125 MHz, CDCl_3_) *δ* 38.79, 43.27, 55.25, 110.62, 121.02, 123.56, 127.18, 127.26, 128.49, 128.84, 131.3 138.54, 157.14, 171.15; HR-MS (ESI^+^) calcd. for C_16_H_18_NO_2_ [M + Na]^+^: 256.1338. Found 256.1342.

Compound **3i**: White solid (470.0 mg, 92.2%); mp 65–67°C; ^1^H NMR (500 MHz, CDCl_3)_ *δ* 7.33–7.42 (m, 6H), 6.96–7.02 (m, 3H), 5.91 (brs, 1H), 4.56–4.57 (d, *J* = 5.7 Hz, 2H), 3.94 (s, 3H), 3.76 (s, 2H); ^13^C NMR (125 MHz, CDCl_3_) *δ* 43.50, 43.82, 55.17, 112.92, 114.94, 121.66, 127.37, 127.43, 128.60, 130.04, 136.20, 138.12, 159.99, 170.70; HR-MS (ESI^+^) calcd. for C_16_H_18_NO_2_ [M + H]^+^: 256.1338. Found 256.1337.

Compound **3j**: White solid (487.6 mg, 97.1%); mp 122–124°C; ^1^H NMR (500 MHz, CDCl_3_) *δ* 7.18–7.32 (m, 8H), 6.87–6.88 (m, 2H), 5.67 (brs, 1H), 4.40–4.41 (d, *J* = 5.8 Hz, 2H), 3.8 (s, 3H), 3.6 (s, 2H); HR-MS (ESI^+^) calcd. for C_16_H_18_NO_2_ [M + H]^+^: 256.1338. Found 256.1339.

^1^H data were consistent with those reported in the literature [35].

Compound **3k**: White solid (267.4 mg, 60.1%); mp 98–100°C; ^1^H NMR (500 MHz, CDCl_3_) *δ* 7.30–7.43 (m, 10H), 5.56 (brs, 1H), 4.36–4.37 (d, *J* = 5.9 Hz, 2H), 3.61 (s, 2H), 2.24 (s, 3H); ^13^C NMR (125 MHz, CDCl_3_) *δ* 19.45, 41.79, 43.43, 126.61, 127.36 (3C), 127.83, 128.59 (2C), 130.48, 130.79, 133.18, 137.17, 138.18, 170.64; HR-MS (ESI^+^) calcd. for C_16_H_18_NO [M + H]^+^: 240.1388. Found 240.1386.

Compound **3l**: White solid (369.8 mg, 80.4%); mp 71–72°C; ^1^H NMR (500 MHz, CDCl_3_) *δ* 7.01–7.26 (m, 10H), 6.01 (brs, 1H), 4.34–4.35 (d, *J* = 5.8 Hz, 2H), 3.52 (s, 2H), 2.30 (s, 3H); ^13^C NMR (125 MHz, CDCl_3_) *δ* 21.31, 43.48, 43.67, 126.39, 127.44 (3C), 128.07, 128.59 (2C), 128.86, 130.16, 134.71, 138.22, 138.68, 171.04; HR-MS (ESI^+^) calcd. for C_16_H_18_NO [M + H]^+^: 240.1388. Found 240.1388.

Compound **3m**: White solid (360.2 mg, 90.3%); mp 133–135°C; ^1^H NMR (500 MHz, CDCl_3_) *δ* 7.16–7.32 (m, 10H), 5.69 (brs, 1H), 4.41–4.42 (d, *J* = 5.7 Hz, 2H), 3.61 (s, 2H), 2.34 (s, 3H); ^13^C NMR (125 MHz, CDCl_3_) *δ* 21.02, 43.35, 43.48, 127.42 (3C), 128.58 (2C), 129.31 (2C), 129.70 (2C), 131.61, 137.04, 138.17, 171.12; HR-MS (ESI^+^) calcd. for C_16_H_17_NONa [M + Na]^+^: 262.1208. Found 262.1212.

Compound **3n**: Pale yellow solid (440.4 mg, 90.4%); mp 126–128°C; ^1^H NMR (500 MHz, CDCl_3_) *δ* 7.41–7.47 (m, 6H), 7.28–7.32 (m, 2H), 7.11–7.20 (m, 2H), 5.81 (brs, 1H), 4.52–4.54 (d, *J* = 5.8 Hz, 2H), 3.64–3.66 (d, *J* = 5.8 Hz, 2H); ^13^C NMR (125 MHz, CDCl_3_) *δ* 43.79, 43.72, 115.33, 115.50, 127.38, 129.02 (2C), 129.36 (2C), 133.95, 134.69, 161.07, 163.03, 170.88; HR-MS (ESI^+^) calcd. for C_15_H_15_NOF [M + H]^+^: 244.1138. Found 244.1143.

Compound **3o**: Light yellow solid (756.5 mg, 85.0%); mp 139–141°C; ^1^H NMR (500 MHz, CDCl_3_) *δ* 6.68–7.47 (m, 13H), 5.91 (brs, 1H), 4.41–4.43 (d, *J* = 5.9 Hz, 2H), 3.78 (s, 3H), 3.71 (s, 2H), 2.37 (s, 3H); HR-MS (ESI^+^) calcd. for C_26_H_23_N_2_O_3_NaCl [M+Na]^+^: 469.1295. Found 469.1290.

^1^H data were consistent with those reported in the literature [18].

Compound **3p**: Light yellow solid (590.0 mg, 50.8%); mp 123–125°C; ^1^H NMR (500 MHz, CDCl_3_), *δ* 7.16–7.32 (m, 9H), 6.86–6.88 (d, *J* = 5.0 Hz, 2H), 5.83–5.85 (d, *J* = 6.5 Hz, 1H), 4.83–4.85 (d, *J* = 7.5 Hz, 1H), 3.64–3.69 (d, *J* = 22.5 Hz, 3H), 3.54–3.55 (d, *J* = 1.5 Hz, 2H), 2.97–3.08 (m, 2H); HR-MS (ESI^+^) calcd. for C_26_H_23_N_2_O_3_NaCl [M+Na]^+^: 320.1263. Found 320.1263.

^1^H data were consistent with those reported in the literature [36].

Compound **3r**: White solid (120.0 mg, 28.6%); mp 133–134°C; ^1^H NMR (500 MHz, CDCl_3_) *δ* 7.79–7.80 (d, *J* = 5.0 Hz, 2H), 7.26–7.52 (m, 9H), 6.43 (brs, 1H), 4.65–4.66 (d, *J* = 5.0 Hz, 2H); HR-MS (ESI^+^) calcd. for C_14_H_14_NO [M + H]^+^: 212.1076. Found 212.1075.

^1^H data were consistent with those reported in the literature [34].

Compound **3s**: White solid (260.0 mg, 68.4%); mp 121–123°C; ^1^H NMR (500 MHz, CDCl_3_) *δ* 7.26–7.36 (m, 6H), 5.68 (brs, 1H), 4.44–4.45 (d, *J* = 5.6 Hz, 2H), 2.20–2.24 (m, 2H), 1.62–1.68 (m, 2H), 1.33–1.40 (m, 2H), 0.84–0.94 (m, 3H); HR-MS (ESI^+^) calcd. for C_12_H_18_NO [M + H]^+^: 192.1385. Found 192.1388.

^1^H data were consistent with those reported in the literature [37].

Compound **3t**: White solid (340.1 mg, 77.6%); mp 156–158°C; ^1^H NMR (500 MHz, CDCl_3_) *δ* 7.26–7.35 (m, 5H), 5.74 (brs, 1H), 4.44–4.45 (d, *J* = 5.7 Hz, 2H), 2.20–2.23 (m, 2H), 1.62–1.66 (m, 2H), 1.26–1.34 (m, 6H), 0.86–0.88 (m, 3H); ^13^C NMR (125 MHz, CDCl_3_) *δ* 14.00, 22.46, 25.74, 28.92, 31.48, 36.54, 43.76, 127.25, 127.61 (2C), 128.51 (2C), 138.44, 173.60; HR-MS (ESI^+^) calcd. for C_14_H_22_NO [M + H]^+^: 220.1703. Found 220.1701.

Compound **3u**: White solid (399.6 mg, 80.9%); mp 137–139°C; ^1^H NMR (500 MHz, CDCl_3_) *δ* 7.26–7.35 (m, 6H), 5.69 (brs, 1H), 4.44–4.52 (d, *J* = 4.1 Hz, 2H), 2.19–2.23 (m, 2H), 1.64–1.66 (m, 2H), 1.26 (brs, 10H), 0.86–0.88 (m, 3H); ^13^C NMR (125 MHz, CDCl_3_) *δ* 14.06, 22.59, 25.76, 29.11,29.26, 29.64, 31.76, 36.64, 43.48, 127.35, 127.69 (2C), 128.58 (2C), 138.34, 173.44; HR-MS (ESI^+^) calcd. for C_16_H_26_NO [M + H]^+^: 248.2014. Found 248.2014.

Compound **3v**: White solid (580.1 mg, 91.3%); mp 140–142°C; ^1^H NMR (500 MHz, CDCl_3_) *δ* 7.26–7.35 (m, 6H), 5.67 (brs, 1H), 4.44–4.45 (d, *J* = 5.6 Hz, 2H), 2.33–2.36 (m, 2H), 2.19–2.23 (m, 2H), 1.25 (brs, 20H), 0.84–0.89 (m, 3H); HR-MS (ESI^+^) calcd. for C_21_H_36_NO [M + H]^+^: 318.2795. Found 318.2797.

^1^H data were consistent with those reported in the literature [38].

## 4. Conclusion

In summary, we have developed eco-friendly and efficient direct amidation of benzylamine and phenylacetic acid derivatives in the presence of 10 mol% NiCl_2_ as catalyst without any drying agent. The product was easily purified and the catalyst was easily recycled. The catalyst can be recycled three times without loss of activity.

## Supplementary Material

Electronic Supplementary Material (ESI) for Royal Society Open Science.

## Data accessibility

This article contains no additional data.
