# Supplementary material for: Direct amidation of non-activated phenylacetic acid and benzylamine derivatives catalysed by NiCl2
Source: R Soc Open Sci. 2018 Feb 21;5(2):171870. doi: 10.1098/rsos.171870 (PMC5830780; doi:10.1098/rsos.171870)
Supplement: Electronic Supplementary Material (ESI) for Royal Society Open Science. [file rsos171870supp1.doc]

**Electronic Supplementary Material (ESI)**

**for Royal Society Open Science.**

Supplementary Material

for

**A Novel and Highly Efficient Esterification Process using Triphenylphosphine Oxide with Oxalyl chloride**

Ming-Zhu Jia, Xiao-Ling Sun

School of Chemical and Environment Engineering, Shanghai Institute of Technology, 201418, Shanghai, China

Xiaolingsun 1@msn.com

NMR spectra of products in Table 3 2-17

**NMR spectra of products**

**Benzyl benzoate:** ^1^H-NMR (CDCl_3_, δppm, 500 MHz)





^13^C-NMR (CDCl_3_, δppm, 125 MHz)





^1^H-NMR (CDCl_3_, δppm, 500 MHz)



^13^C-NMR (CDCl_3_, δppm, 125 MHz)





^1^H-NMR (CDCl_3_, δppm, 500 MHz)





^13^C-NMR (CDCl_3_, δppm, 125 MHz)





^1^H-NMR (CDCl_3_, δppm, 500 MHz)





^13^C-NMR (CDCl_3_, δppm, 125 MHz)





^1^H-NMR (CDCl_3_, δppm, 500 MHz)





^13^C-NMR (CDCl_3_, δppm, 125 MHz)





^1^H-NMR (CDCl_3_, δppm, 500 MHz)





^13^C-NMR (CDCl_3_, δppm, 125 MHz)





^1^H-NMR (CDCl_3_, δppm, 500 MHz)





^13^C-NMR (CDCl_3_, δppm, 125 MHz)





^1^H-NMR (CDCl_3_, ppm, 500 MHz)





^13^C-NMR (CDCl_3_, δppm, 125 MHz)





^1^H-NMR (CDCl_3_, δppm, 500 MHz)





^13^C-NMR (CDCl_3_, δppm, 125 MHz)





^1^H-NMR (CDCl_3_, δppm, 500 MHz)





^13^C-NMR (CDCl_3_, δppm, 125 MHz)





^1^H-NMR (CDCl_3_, δppm, 500 MHz)





^13^C-NMR (CDCl_3_, δppm, 125 MHz)





^1^H-NMR (CDCl_3_, δppm, 500 MHz)





^13^C-NMR (CDCl_3_, δppm, 125 MHz)





^1^H-NMR (CDCl_3_, δppm, 500 MHz)





^13^C-NMR (CDCl_3_, δppm, 125 MHz)





^1^H-NMR (CDCl_3_, δppm, 500 MHz)





^13^C-NMR (CDCl_3_, δppm, 125 MHz)





^1^H-NMR (CDCl_3_, δppm, 500 MHz)





^13^C-NMR (CDCl_3_, δppm, 125 MHz)





^1^H-NMR (CDCl_3_, δppm, 500 MHz)





^13^C-NMR (CDCl_3_, δppm, 125 MHz)
